# Outcome after treatment of distal radius fractures in the elderly using the IlluminOss^®^ System

**DOI:** 10.1007/s00068-019-01289-w

**Published:** 2020-01-16

**Authors:** Guido W. Van Oijen, Paul A. Vegt, Tjebbe Hagenaars, Esther M. M. Van Lieshout, Michael H. J. Verhofstad

**Affiliations:** 1 Trauma Research Unit Department of Surgery, Erasmus MC, University Medical Center Rotterdam, P.O. Box 2040, 3000 CA Rotterdam, The Netherlands; 2 Department of Surgery, Albert Schweitzer Hospital, Dordrecht, The Netherlands

**Keywords:** Distal radius fracture, Elderly, IlluminOss, Intramedullary, Minimally invasive, Retrospective

## Abstract

**Purpose:**

Distal radius fractures are very common and account for approximately 17% of all fractures treated. Multiple treatment methods are available to treat these fractures, both operative and nonoperative. This study aimed at evaluating the functional and clinical outcomes after treatment of distal radius fractures with the IlluminOss^®^ System in adult patients.

**Methods:**

A retrospective case series was performed in a single-level two-trauma center. All consecutive adult patients with a distal radius fracture, treated with the IlluminOss^®^ System between 01 August 2012 and 15 August 2015, were included in this study. Baseline patient characteristics and clinical data were retrospectively extracted from the medical records. Radial inclination, volar/dorsal tilt, ulnar variance, and radial length were measured on the latest available standard radiographs. In addition, patients were prospectively subjected to physical examination and were asked to complete the Disabilities of the Arm, Shoulder, and Hand, Patient-Rated Wrist Evaluation, and Short Form-36 questionnaires.

**Results:**

Twenty-six patients with 31 distal radius fractures were included. The median age at time of trauma was 77 years and 96% were females. Five patients developed a total of seven complications. Due to persisting pain one reoperation was performed, removing a small prominent part of the implant. Both patient-reported outcome scores and radiographic results were good to excellent.

**Conclusions:**

The IlluminOss^®^ System is a feasible option to treat distal radius fractures with seemingly good clinical and functional outcome. One out of seven complications required surgical intervention. These outcomes justify more detailed prospective research.

## Introduction

Fractures of the distal radius account for an estimated 17% of all fractures treated in US Emergency Departments and make up one of the most common osteoporotic fractures [1, 2]. Because of the osteoporotic character of distal radius fractures, elderly women are most likely to sustain a distal radius fracture with a female:male ratio of about 3:1 and a peak incidence between 60 and 69 years of age [3–5]. The absolute number of hospitalizations due to these fractures in The Netherlands in patients aged 50 years and older increased from 877 in 1997 to 2912 in 2009 and a further increase is expected [3, 4]. This will bring a concomitant increase in demand for health care resources [6, 7].

**Fig. 1:**
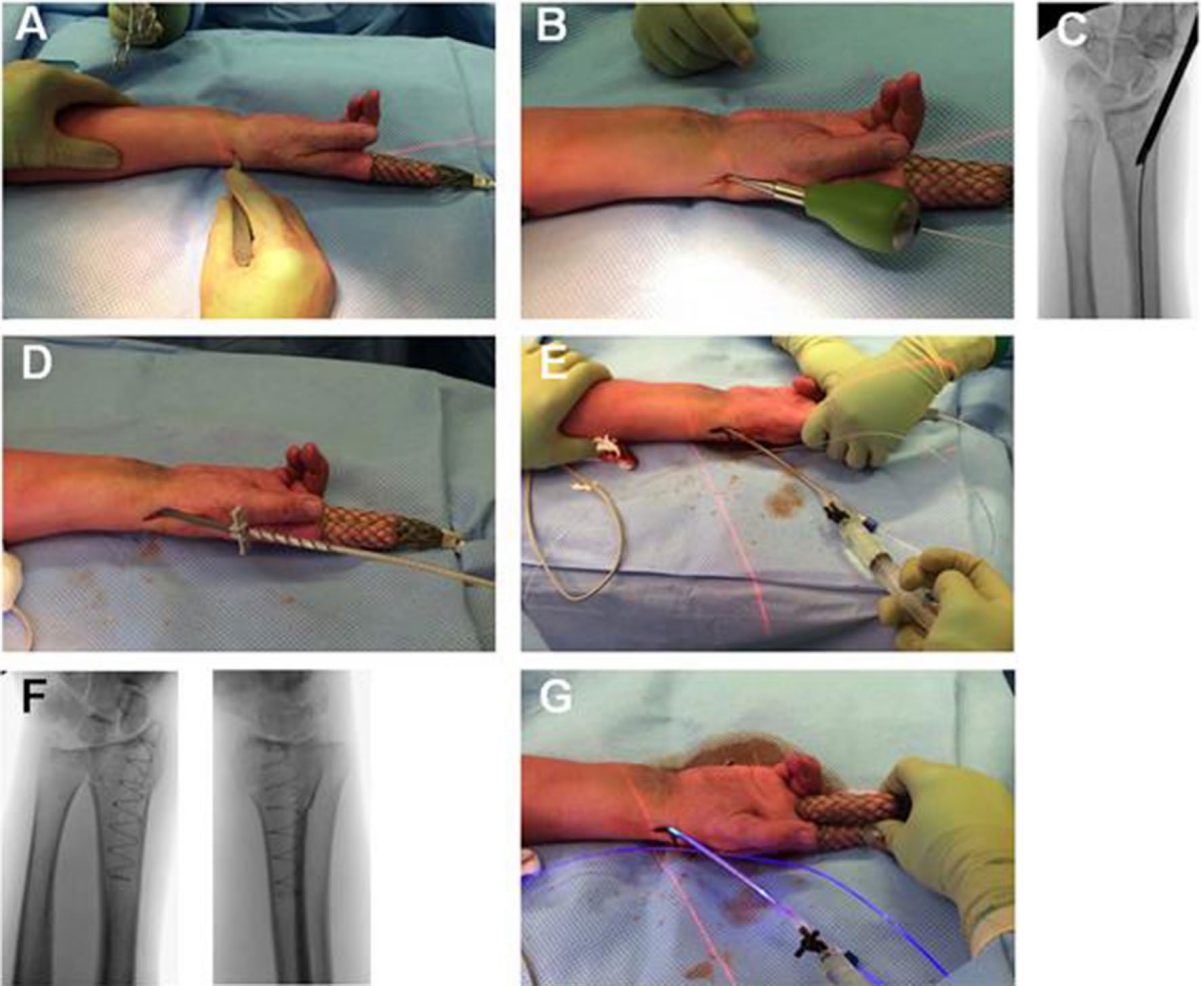
Using the IlluminOss^®^ System for fixating a distal radius fracture. **a** 1.5–2.0 cm incision over the radial styloid process, between the first and second extensor compartment to reach the periosteum. The branches of the superficial radial nerve are protected. **b** Access to the metaphyseal bone and medullary canal and insertion of a 1.5 mm guide-wire. **c** Correct position is verified by intra-operative fluoroscopy. **d** Flexible balloon catheter is placed intramedullary over the guide-wire spanning the fracture. **e** Infusion of liquid monomeric material and expansion of the balloon conforming to the patient’s unique medullary canal. **f** Verification of adequate fracture reduction, correct balloon position, and balloon expansion. **g** Polymerization (hardening) of the infused monomer by applying visible (436 nm) light, creating a patient-conforming intramedullary implant

Fractures of the distal radius can be treated operatively or non-operatively. Operative treatment can be done by external fixation or internal fixation using pins, screws, plates, or intramedullary nails. The IlluminOss^®^ System (IlluminOss^®^ Medical, East Providence, RI, USA) is a minimally invasive, patient-conforming, intraosseous fracture stabilization system [8]. A small incision of approximately 1.5–2 cm is required to insert an inflatable balloon into the medullary canal. The balloon, which spans the fracture, is infused with a monomer that polymerizes and hardens by applying blue light. This results in a stable and patient-conformed implant that can provide longitudinal strength and rotational stability (Fig. 1). The minimally invasive nature of the procedure and immediate start of functional after-treatment without plaster cast immobilization are considered the main strengths of this device [8].

We expect that treating patients with the IlluminOss^®^ System will result in excellent recovery of function; however, there are no literature data to confirm this. Therefore, the aim of this study was to evaluate functional and clinical outcomes after treatment of distal radius fractures with the IlluminOss^®^ System in adult patients.

## Materials and methods

### Patients and setting

All adult patients with a distal radius fracture, treated with the IlluminOss^®^ System between 01 August 2012 and 15 August 2015 at a level II trauma center, were included in this retrospective study. Patients with both extra- and intraarticular fractures were eligible. Patient selection for IlluminOss^®^ treatment was done by the treating surgeon (PAV). The main selection criteria were the need and ability for fast return to daily activities and fracture type. Patients with a pathological or open fracture (i.e., Gustilo type II or III) were excluded. The local Medical Research Ethics Committee has given a waiver for this study. All patients participating in the follow-up measurement provided informed consent.

### Data collection and outcomes assessment

The medical files of all patients were reviewed and the following patient-related parameters were retrieved: age, gender, comorbidities, ASA-classification, trauma mechanism, and additional injuries. The intervention-related variables recorded were: time between injury and surgery, length and diameter of polyethylene terephthalate (PET) balloon used, peroperative complications (i.e., reduction problems, technical device-related problems, or iatrogenic damage), duration of surgery (i.e., total time of operation room use and net time for surgery). Finally, hospital length of stay, discharge destination, and physical therapy details were collected from the medical files.

Radiographic evaluation was done in duplicate (GWVO and TH). Fractures were classified according to the AO/OTA-classification [9]. Radial inclination, volar/dorsal tilt, ulnar variance, and radial length were measured digitally in the Picture Archiving and Communication System (PACS) on the latest available standard posteroanterior and lateral wrist radiographs. Fracture consolidation and the Lidstrom score were also determined from these radiographs [10]. Measurements were averaged. Discrepancies were resolved by consensus.

The primary outcome measure was infection rate. Infectious complications were divided into superficial (i.e., minor or treated with oral antibiotics only) and deep (i.e., major or requiring surgical intervention, readmission, or intravenous antibiotics) by applying the criteria of the Centers for Disease Control and Prevention [11].

Patients were invited to visit the outpatient department. A trained researcher (GWVO) measured range of motion (ROM) with a goniometer, and grip strength with a Jamar^®^ Hydraulic Hand Dynamometer. Measurements were standardized. Patients were also asked to complete the following questionnaires: disabilities of the arm, shoulder, and hand (DASH), patient-rated wrist evaluation (PRWE), and the level of pain during specified activities (Numeric rating scale, NRS), health-related quality of life (Short-form-36, SF-36). An additional questionnaire asked about the time to regaining independence in activities of daily living (ADL) and the use of physical therapy.

### Data analysis

Descriptive analysis was performed using the Statistical Package for the Social Sciences (SPSS) version 21.0 (SPSS, Chicago, Ill., USA). Data are reported following the STROBE guidelines [12]. Continuous data all deviated from the Normal distribution and are, therefore, shown as median with quartiles. Categorical data are shown as number with percentage. Spearman’s rank correlation tests were performed in order to determine the correlation of the Lidstrom score with the DASH, PRWE, flexion–extension arch, deviation arch, and pronation–supination arch.

## Results

### Patient and intervention characteristics

Approximately 1500 patients (> 60 years old) were treated for a wrist fracture (ICD-10 code S52.5) between 01 August 2012 and 15 August 2015 in this level II trauma center. In about a quarter of these patients this concerned an extra-articular distal radius fracture. Only one of the four trauma surgeons in this trauma center treated patients with the IlluminOss^®^ System and offered this as an experimental treatment option. This resulted in a total of 26 patients with 31 distal radius fractures that were treated with the IlluminOss^®^ System. Eighteen patients with 21 fractures underwent physical examination and completed the questionnaires at a median of 21 (*P*_25_–*P*_75_ 18–36) months after fracture. Four patients had deceased and four others wished not to participate. Table 1 shows the patient characteristics of the entire study population. The median age at time of trauma was 77 years and the majority of patients (*N* = 25; 96%) females. Most patients (*N* = 22; 85%) had an ASA-score I or II. A fall from standing height was the cause of the injury in 25 (96%) patients.

**Table 1:** Patient characteristics

	All patients, *N* = 26	Unilateral fractures, *N* = 20^a^	Bilateral fractures, *N* = 10
Age (years)	77 (70–85)		
Female gender	25 (96%)		
ASA-classification			
1	6 (23%)		
2	16 (62%)		
3	4 (15%)		
4	0 (0%)		
AO-classification^a^			
23-A2	12 (40%)	5 (25%)	7 (70%)
23-A3	14 (47%)	11 (55%)	3 (30%)
23-C2	3 (10%)	3 (15%)	
23-C3	1 (3%)	1 (5%)	
Right-side affected	17 (55%)		
Trauma mechanism			
Simple fall	25 (96%)		
Bike crash	1 (4%)		
Concomitant injuries	1		

Data are shown as median (*P*_25_–*P*_75_) or as *N* (%)

^a^Radiographs of one patient were not retrieved

The intervention characteristics are outlined in Table 2. In 29 fractures, a single incision at the radial styloid of 1.5 cm was made for the introduction of the IlluminOss^®^ balloon. In two cases (7%) an extra (dorsal) incision was needed to acquire an adequate fracture reduction. The median surgical time, excluding anesthesia was 47 min for unilateral fractures and 87 min for bilateral fractures. After a median admission time of 1 day, 23 (92%) patients were discharged to their home.

**Table 2:** Intervention characteristics

	All patients, *N* = 26		All fractures, *N* = 31	
	*N*		*N*	
Days between ER presentation and surgery	26	9 (6–12)		
Surgical time excl. anesthesia (min)	26	50 (41–87)		
Unilateral	21	47 (37–61)		
Bilateral	5	87 (58–93)		
Balloon length			31	
160 mm				27 (87%)
180 mm				4 (13%)
Balloon diameter			31	
11 mm				30 (97%)
9 mm				1 (3%)
Peroperative complication			31	
Reduction difficulty requiring extra incision				2 (7%)
Admission time (days)	26	1 (1–2)		
Discharge destination	26			
Home		23 (92%)		
Nursing home		1 (4%)		
Rehabilitation center		1 (4%)		
Unknown		1 (4%)		

Data are shown as median (*P*_25_–*P*_75_) or as *N* (%)

### Complications

**Fig. 2:**
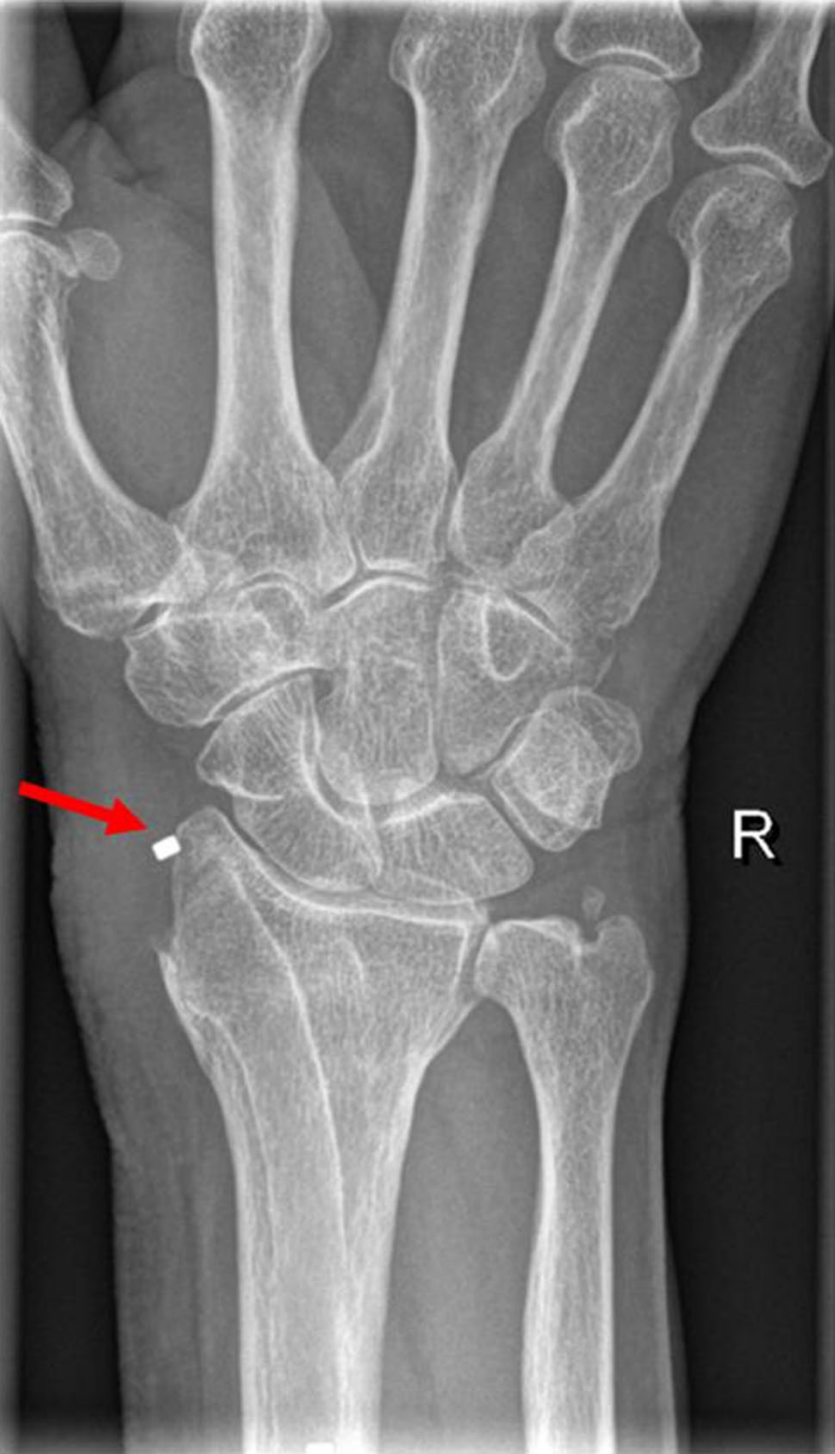
PA-radiograph at one year and 4 months after primary surgery. A small part of the implant is sticking out just proximal of the radial styloid, access point to the intramedullary canal during primary operation

Five patients developed a total of seven complications after surgery (Table 3). Two superficial infections were treated successfully with antibiotics. Two patients had neurapraxia of the superficial radial nerve, which was self-limiting. One patient reported persisting ulnar pain at 9 months after surgery requiring oral pain medication. One patient had pain around the radial styloid, which is the entrance point of the implant. At both physical and radiographic examination the implant appeared proud, irritating soft tissues (Fig. 2). Removing this part of the implant resulted in a complete relief of complaints.

**Table 3:** Complications

	All fractures, *N* = 31	
	*N*	
Complications	31	7 (22%)
Superficial surgical site infection		2 (6%)
Neurapraxia radial superficial nerve		2 (6%)
CRPS		1 (3%)
Persisting ulnar pain		1 (3%)
Implant-related pain		1 (3%)

Data are shown as *N* (%)

### Radiographic results

Out of the 31 fractures, 30 fractures had at least one postoperative radiograph. Twenty patients had radiographic evaluation beyond 6 weeks after surgery. The median time to last X-ray was 177 days after surgery. Twenty-three fractures (77%) showed good or excellent fracture reduction according to the Lidstrom criteria (Table 4). Twenty-three (77%) fractures showed a dorsal angulation with a median of 3 (*P*_25_–*P*_75_ 1–8) of the fracture fragment at the last available radiograph. This was the most common deformity that was responsible for lower Lidstrom scores. No signs of late secondary loss of reduction were seen.

**Table 4:** Radiographic results

	All fractures, *N* = 30^a^
Radial inclination (°)	23.5 (17–27)
Ulnar variance (mm)	3 (1–5)
Volar angulation (°) (7 fractures)	5 (1–8)
Dorsal angulation (°) (23 fractures)	3 (1–8)
Lidström classification	
Excellent	6 (20%)
Good	17 (57%)
Fair	5 (17%)
Poor	2 (7%)

Data are shown as median (*P*_25_–*P*_75_) or as *N* (%)

^a^Radiographs of one patient were not retrieved

The other seven fractures showed a volar angulation with a median of 5 (*P*_25_–*P*_75_ 1–8). All patients with radiographs beyond 6 weeks after surgery (*N* = 20), with a median time to last X-ray of 177 days after surgery, showed radiographic healing. Apart from increased sclerosis around the implant, no other bone abnormalities (i.e. degeneration or lysis) were seen. Although the exact borders of the implant are not clearly visible on conventional X-rays, during follow-up we saw no lucent zones (indicating loosening) within the bone marrow.

### Functional outcome

Table 5 shows the results of the questionnaires. Both the DASH score and the PRWE total score had a median of nine points. The median pain score during specified activities was zero.

**Table 5:** Functional outcome: patient-rated outcome measures

	All patients, *N* = 18
DASH score	9 (1–26)
PRWE total score	9 (0–25)
PRWE pain score	1 (0–17)
PRWE functional score	5 (0–17)
Pain at rest^a^	0 (0–0)
Pain at work^a^	0 (0–2)
Pain during housekeeping activities^a^	0 (0–2)
SF-36 total score	102 (92–111)
SF-36 physical component score	46 (33–54)
SF-36 mental component score	60 (54–63)

Data are shown as median (*P*_25_–*P*_75_)

^a^Only patients who completed the set of questionnaires

Patients with a unilateral fracture showed 18% loss of range of deviation and a 20% loss in grip strength in the affected side as compared to the uninjured wrist (Table 6). Both the flexion–extension and pronation-supination restriction were less than 10%. This was in line with statements of the treating surgeon that patients achieved full range of motion. A statistically significant correlation was found between the Lidstrom score and flexion–extension arc with a Spearman’s rho of − 0.437 (*p* = 0.047). Correlations of the Lidstrom score with other functional outcomes (i.e., DASH score, PRWE score, deviation arc, and pro-supination arc) did not reach statistical significance (Spearman’s rho from 0.2387 to 0.313).

**Table 6:** Functional outcome: clinical measures

	All fractures, *N* = 23
Loss of ROM (degrees)	
Flexion–extension	5 (− 6–26) = 8%
Ulnar deviation–radial deviation	8 (1–24) = 18%
Pronation–supination	1 (− 7–22) = 0.1%
Loss of grip strength (kg)	2 (0–7) = 20%

^a^Only patients who underwent physical examination

## Discussion

The aim of this study was to evaluate outcome of treatment of distal radius fractures with a new patient-conforming device, the IlluminOss^®^ system. The essence of the system is that after percutaneous introduction a final indirect fracture reduction can be performed and subsequently the monomer is hardened to provide stability. Clinical and functional outcome in terms of ROM, DASH and PRWE scores at 21-month follow-up were good, as was radiological outcome. Seven complications developed in 31 fractures, of which one required a small surgical intervention.

The retrospective design could be considered a limitation of the current study. With only 31 fractures included and a considerable rate of missing data for specific outcomes, the sample size was relatively small. Increasing the population was not feasible due to start of a prospective study [13]. The non-randomized design may also be considered a limitation. However, given the large number of publications on outcome after treatment of distal radius fractures, we preferred to compare the results with published data. Follow-up visits and medical record notes were not standardized. The course of improvement of function, ADL independence, and the amount of physical therapy provided were not reliably documented and, therefore, not analyzed. Moreover, as any retrospective study selection bias likely occurred since the decision to use the IlluminOss^®^ system depended on the preference of the attending surgeon as well as logistical considerations. This may explain why the study population was relatively older and more often female patients than expected from recent literature [14–16].

The DASH score (9 points) and PRWE score (9 points) seem slightly lower than published for other fixation methods. Currently, no patient-reported outcome scores are available for treatment of distal radius fractures with the IlluminOss^®^ System. A previous study of Costa et al*.* reported a mean DASH of 13.0 at 1 year after volar plate fixation and 16.2 after percutaneous fixation with K-wires [17]. Mean PRWE scores were 13.9 after volar plate fixation and 15.3 after percutaneous fixation with K-wires [17]. This may suggest that the patients in the current study experienced less disability and a better function. An explanation for this could be the fact that the IlluminOss^®^ system is a percutaneous, patient-conforming fracture stabilization system. However, a longer follow-up duration in the current study (21 months) may also influence the difference in scores. Previous studies suggest that further improvement of functional scores can be expected after 1 year [18]. Due to the retrospective and non-comparative design, no conclusions can be drawn with regard to the course of functional improvement. The strong feeling is that patients treated with the IlluminOss^®^ system experience a faster functional recovery than those with non-operative treatment or open reduction and plate fixation. Therefore, a prospective study was started [13].

In this study we found a complication rate of 22%, where in one case a re-operation was performed, approximately 2 years after primary surgery. Literature provides a large diversity of complications and complication rates for K-wire fixation (6.5–28%), plate fixation (3.2–36%), and intramedullary nailing (18–36%) [17, 19–26]. In this study the percentage of complications was within these ranges. Since every fixation method has its own specific complications, these percentages cannot easily be compared.

Good or excellent radiographic results after surgical treatment of distal radius fractures have been reported frequently [22, 25, 27–29]. Brennan et al. found superior radiological outcome in favor of volar plate fixation, when comparing it to percutaneous K-wire fixation [28]. The radial inclination was 22.1 and 21.3, the ulnar variance was − 0.5 and 0.1, and the volar tilt was 4.2 and 1.7. These values correspond well with previously published studies [22, 29]. The current study showed similar values except for the volar tilt, where the majority of the fractures showed a dorsal tilt at final follow-up. From the available radiographs, it was not possible to judge if this was due to primary reduction errors or to secondary collapse, because for most patients no radiographs within the first week after surgery were available.

During follow-up of our study subjects, no removal of implants was necessary. If necessary (e.g. due to deep infection, loss of reduction or re-fracture), the implant can be removed in three ways: (1) by simple grabbing and pulling. Especially if a loosened implant is the likely cause of secondary dislocation, it will be easy, (2) after pre-drilling a hole in the balloon, a Steinmann’s pin, threaded guidewire, or screw can be inserted to extract the implant, with or without the use of the IlluminOss^®^ removal slap hammer, (3) an ultrasonic 63 kHz implant-pulverizing device is available (IlluminOss^®^ Medical, East Providence, RI, USA). This device pulverizes the cured polymer, but leaves the balloon intact. After that, the emptied balloon can be easily extracted.

In conclusion, this study showed good functional and radiographic outcomes, with similar complication rates as in recent literature. A larger and prospective study is needed in order to confirm this and to provide insight into the timing of functional recovery.
